# Influence of Selected Antidepressants on the Ciliated Protozoan *Spirostomum ambiguum*: Toxicity, Bioaccumulation, and Biotransformation Products

**DOI:** 10.3390/molecules25071476

**Published:** 2020-03-25

**Authors:** Grzegorz Nałęcz-Jawecki, Milena Wawryniuk, Joanna Giebułtowicz, Adam Olkowski, Agata Drobniewska

**Affiliations:** 1 Department of Environmental Health Sciences, Faculty of Pharmacy, Medical University of Warsaw, ul. Banacha 1, 02-097 Warszawa, Poland; mwawryniuk@wum.edu.pl (M.W.); olek_adam@interia.pl (A.O.); 2 Department of Bioanalysis and Drugs Analysis, Faculty of Pharmacy, Medical University of Warsaw, ul. Banacha 1, 02-097 Warszawa, Poland; jgiebultowicz@wum.edu.pl

**Keywords:** Spirotox, fluoxetine, sertraline, paroxetine, mianserin, pharmaceuticals in the environment

## Abstract

The present study aimed to evaluate the effect of the most common antidepressants on aquatic protozoa. *Spirostomum ambiguum* was used as the model protozoan. The biological activity of four antidepressants, namely fluoxetine, sertraline, paroxetine, and mianserin, toward *S. ambiguum* was evaluated. Sertraline was found to be the most toxic drug with EC_50_ values of 0.2 to 0.7 mg/L. The toxicity of the antidepressants depended on the pH of the medium and was the highest in alkaline conditions. Sertraline was also the most bioaccumulating compound tested, followed by mianserin. Slow depuration was observed after transferring the protozoa from the drug solutions to a fresh medium, which indicated possible lysosomotropism of the tested antidepressants in the protozoa. The biotransformation products were identified using a high-resolution mass spectrometer after two days of incubation of the protozoa with the tested antidepressants. Four to six potential biotransformation products were observed in the aqueous phase, while no metabolites were detected in the protozoan cells. Because of the low abundance of metabolites in the medium, their structure was not determined.

## 1. Introduction

Protozoa play an important role in the aquatic food web as primary consumers. They are common in surface waters and activated sludge in waste-water treatment plants (WWTP), where they feed on bacteria and may ingest pollutants directly from water. *Spirostomum ambiguum* is one of the largest ciliated protozoa with long generation time of about 70 h. It tolerates pH changes from 5.5 to 8.0, can be cultured in laboratory, and stored in an inorganic medium for at least eight days [1]. Thus, it is a very convenient organism and has been used in ecotoxicological studies for more than 25 years [1,2,3].

Antidepressants are one of the major group of pharmaceuticals used worldwide. Sertraline, fluoxetine, and paroxetine, belonging to the most commonly used selective serotonin re-uptake inhibitors (SSRIs), were ranked 14, 31, and 68, respectively, on the top 300 best-selling drugs in 2020 with 38.3, 21.9, and 11.7 million prescriptions, respectively, in the U.S. in 2017 (www.clincalc.com, accessed: 7 February 2020). Mianserin is an atypical, tetracyclic antidepressant used for the treatment of major depressive disorders. Antidepressants, as with other pharmaceutically active compounds (PhACs), are released into freshwaters mainly with waste-water, and they have been detected in effluents, freshwaters, and even drinking waters in many countries [4,5,6,7,8,9]. Mole and Brooks [10] wrote a comprehensive review of the occurrence of SSRIs in the environment. They found that fluoxetine, citalopram, paroxetine, and sertraline, and their main metabolites norfluoxetine and norsertraline were the most commonly detected antidepressants. Their concentrations in influents and effluents were up to several μg/L. SSRIs have been identified not only in water and sediments but also in fish caught in the effluent waste streams and in molluscs and fish tissues commonly consumed by humans [11]. Most of the SSRIs are slowly degraded under the influence of both biotic and abiotic factors, and due to the continuous discharge are called pseudo-persistent contaminants [11].

High biological activity of antidepressants, especially SSRIs, has been reported for algae [12] and crustaceans and fish [11]. In short-term acute toxicity tests, the EC_50_ values ranged from 0.2 to 10 mg/L [11,12]. However, in chronic toxicity assays, the lowest observed effect concentration (LOEC) values were much lower, as low as 0.0136 mg/L for *Pseudokirchneriella subcapitata* [12]. Moreover, SSRIs are considered to be potentially bioaccumulative [13]. They were detected in fish [14,15] and bivalve [16] tissues. Sertraline was the most bioaccumulating compound in the effluent reach stream [17]. Its bioaccumulation factor (BCF) in two benthic organisms *Hydropsyche* sp. and *Erpobdella octoculata* ranged from 920 to 2100 L/kg and was close to the predicted value. There is a lack of knowledge of the response of protozoa to PhACs, probably due to their small size that entails the use of more sophisticated research techniques.

The analysis of the occurrence of PhACs and their human metabolites in the environment has been restricted to compounds with standards available in the market [8,10,18]. With the development of new analytical mass spectrometers, i.e., Quadrupole time of flight (QTOF) working in all ion MS/MS modes and high-resolution Orbitrap™, it has become possible to detect a huge amount of non-target PhAC metabolites and transformation products [18,19] not only of human origin but also of microbial origin. However, neither PhAC nor their metabolites have yet been reported for their effect on protozoa. Some antidepressant metabolites, e.g., norfluoxetine, have even higher biological activity as parent compounds [20]. Thus, to recognize the complete risk of drugs occurring in the aquatic environment, it is important to know both biotic and abiotic transformation of these compounds.

The present study aimed to evaluate the biological activity of four antidepressants, namely fluoxetine, sertraline, paroxetine and mianserin, on the ciliated protozoan *S. ambiguum*. Our comprehensive study included analysis of acute toxicity, bioconcentration, and biotransformation. Acute toxicity was evaluated with the prolonged, seven-day Spirotox assay. As the tested compounds ionize in water solution, three pH levels from the range of the pH of natural freshwaters (6.0, 6.5 and 7.4) were tested, and the relationship between pH and toxicity was discussed. The bioconcentration of the tested pharmaceuticals was analyzed in the six days uptake followed by six days depuration phases, while the biotransformation products were identified after two days of incubation of the protozoa with the tested antidepressants. In the bioconcentration tests, high-performance liquid chromatography (HPLC) with the mass spectrometer detector QTRAP™ was used, while in the biotransformation tests, UPLC with MS/MS Orbitrap™ was applied with post facto analysis performed by Compound Discoverer Software (Thermo Fisher Scientific, Waltham, MA). To the best of our knowledge, this is the first study on the bioaccumulation and possible biotransformation of PhACs by protozoa.

## 2. Results

### 2.1. Toxicity

The protozoan *S. ambiguum* could be stored in an inorganic medium for a long time without losing its viability; thus, the Spirotox test could be prolonged up to seven days. In all tests, the toxic effect percent in the negative control was less than 10%; thus, the results of the tests were valid.

**Figure 1:**
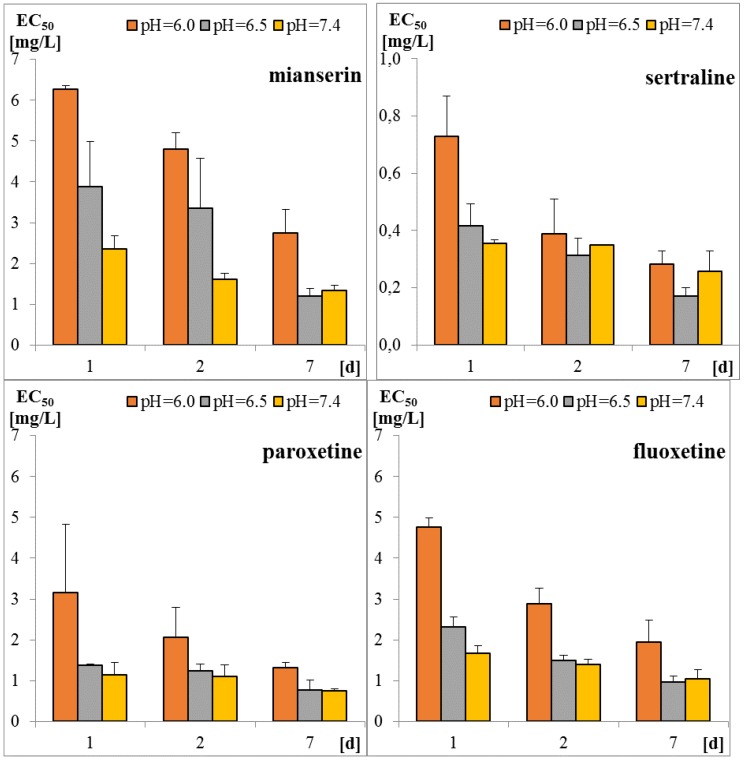
Toxicity of tested antidepressants in Spirotox test after 1, 2, and 7 days incubation (EC_50_ expressed in mg/L).

Sertraline was the most toxic antidepressant in all the tested approaches, with EC_50_ in the range of 0.2–0.7 mg/L (Figure 1 and Table A1). Paroxetine and fluoxetine were three-fold, while mianserin was 10-fold less toxic than sertraline. The tested compounds were acutely toxic to *S. ambiguum*, as the LC_50_ and EC_50_ values were close to each other. This implies that sublethal effects quickly became lethal ones. Moreover, in most cases, the EC_20_ values were less than two times lower than EC_50_ values (Table A1). Only for fluoxetine and mianserin tested at pH lower than seven, the EC_50_ to EC_20_ ratio was higher than two. EC_20_ is a threshold value that indicates the threat to the population of the tested organism. This implies that the EC_50_ value is a good predictive value that can be used to predict the effects of the substances on an entire population. As expected, the toxicity increased with the time of incubation, and the seven-day values were much lower than the one- and the two-day values. The toxicity also depended on the pH of the medium. *S. ambiguum* could be tested in a wide range of pH from 5.5 to 8.0. The toxicity was measured at three pH values 6.0; 6.5 and 7.4 to imitate natural freshwaters. For all tested antidepressants, an increase in toxicity was observed with increasing pH. For SSRIs, the step change can be seen between pH 6.0 and 6.5, while for mianserin, the toxicity increased gradually with the increase in pH, especially after one and two days of incubation. The relationship between toxicity and pH of the medium was previously reported for nitrophenols [21], and to the best of our knowledge, this relationship has not been tested for pharmaceuticals thus far. The toxicity-to-water pH relationship has two consequences. First, the pH of the water should be more strictly defined in the ecotoxicity guidelines to prevent high variability of the results. The present data indicate that the pH shift by only one unit may result in a significant change in toxicity. Second, pH of the water and effluent should be considered in the environmental risk assessment of the ionizable compounds. The tested antidepressants are cationic amphiphilic drugs that ionize in acidic solutions, and the bioavailability of the ionized form of the compound is lower than that of the non-ionized one. For many amphiphilic compounds, the biological activity may be predicted using the pH-dependent water/octanol partition coefficient (log D) instead of log P. Taking into account the whole group of compounds tested there was no correlation between the toxicity of the antidepressants to *S. ambiguum* and lipophilicity expressed by both log P and log D coefficients (Table 1). Thus, their biological activity cannot be explained by the simple non-polar and polar narcosis mechanism of action [22]. The tested drugs inhibit neurotransmitter’s (serotonin) re-uptake in vertebrate’s tissues. Minguez et al. [23] reported the correlation of SSRI toxicity towards *Daphnia* with the log P coefficient. However, they also observed irreversible cell lysis in the abalone hemocytes, probably due to interactions between the drugs and lysosomal membrane phospholipids [23]. As vacuolization was the first symptom of toxicity of the tested compounds in *S. ambiguum*, we expected that such interactions also occur in protozoa and are the main reason of toxic effects.

Antidepressants, especially sertraline, are very potent against parasitic protozoa with IC_50_ of 0.16 mg/L and 0.24 mg/L for *Plasmodium falciparum* and *Trypanosoma brucei rhodosiensis*, respectively, and are considered to be applicable in the treatment of relevant tropical diseases caused by these parasites [24]. Palit and Ali [25] reported high activity of sertraline against another parasite protozoan *Leishmania donovani*. They hypothesized that sertraline induces cell apoptosis by lowering adenosine triphosphate (ATP) levels, resulting in a reduction in oxygen consumption. However, more research is needed to prove this hypothesis and to determine the mode of action of antidepressants towards protozoa.

The protozoan *S. ambiguum* appeared to be comparably sensitive as other organisms used in acute toxicity bioassays. Similar to our results, sertraline was reported to be the most toxic antidepressant to crustaceans with 48-h LC_50_ of 0.12 mg/L for *Ceriodaphnia dubia* [26], 24-h LC_50_ of 0.6 mg/L for *Thamnocephalus platyurus* [27] and 48-h EC_50_ of 0.92 mg/L for *Daphnia magna* [28]. Slightly lower toxicity was reported for fluoxetine, ranging from 0.23 and 0.82 mg/L for *C. dubia* and *D. magna* [12] to 0.85 mg/L for *T. platyurus* [29]. Contrary to the previous two antidepressants, paroxetine was 10-fold less toxic to *D. magna* (6.3 mg/L) [28] than to *C. dubia* (0.58 mg/L) [26]. Very little information is available for mianserin. Wawryniuk et al. [30] reported 24-h LC_50_ of 1.8 mg/L for *T. platyurus*, while Minguez et al. [23] showed 48-h EC_50_ of 7.81 mg/L for *D. magna*. Similar acute toxicity data were reported for fish: 48-h LC_50_ of 0.198 mg/L for fluoxetine towards *Pimephales promelas* [31] and 96-h LC_50_ of 0.38 mg/L for sertraline towards *Oncorhynchus mykiss* [27]. These values are 2–3 orders of magnitude higher than the levels of antidepressants detected in municipal effluents and freshwaters, and therefore, the acute toxicity effect is not expected in the environmental samples.

### 2.2. Bioaccumulation

To evaluate bioaccumulation of the tested antidepressants in protozoa, *S. ambiguum* was incubated with the antidepressants at three concentrations: low (10 μg/L), medium (25 μg/L), and high (100 μg/L) for six days uptake phase, followed by six days depuration phase.

**Figure 2:**
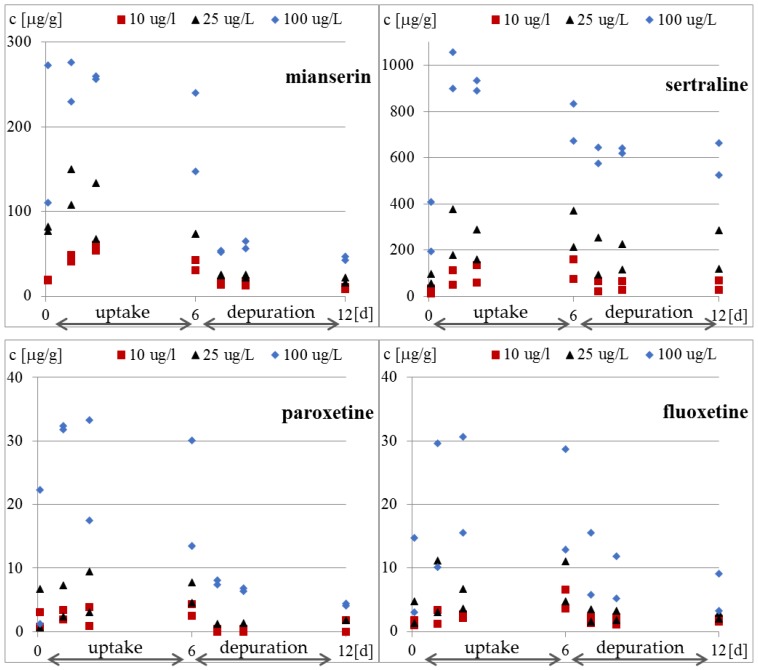
Concentration of tested antidepressants in *S. ambiguum* cells [μg/g].

**Figure 3:**
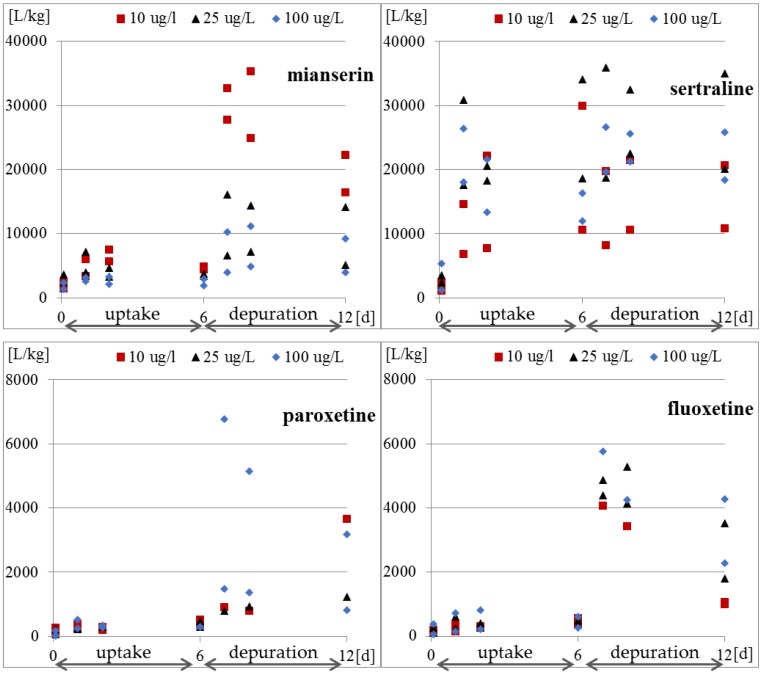
Bioconcentration factor expressed as the ratio of concentration of tested antidepressants in *S. ambiguum* cells to the concentration in water.

Whole-body internal concentrations based on the parent compound were measured. The concentrations of the compounds inside the protozoa and in the medium were determined four times in each research phase. The results of the concentration of the tested antidepressants in *S. ambiguum* cells and in the medium are shown in Figure 2 and Table A2, while the BCF values are presented in Figure 3 and Table A3. From the internal concentration data, it can be concluded that uptake and elimination kinetics vary greatly between the tested pharmaceuticals. *S. ambiguum* accumulated significant amounts of sertraline and mianserin, but different bioaccumulation scenarios were observed in each case and for each drug concentration. The concentration of sertraline in the protozoan cells increased gradually during the uptake phase for low and medium drug concentration. For the highest level tested, the highest sertraline concentration was determined after 24 h, followed by a gradual decrease in its concentration. In the depuration phase, the sertraline intracellular concentration remained at a high level, falling by only 40% of the highest concentration (all tested concentrations). Mianserin reached its highest concentration in *S. ambiguum* cells after two days of incubation. After six days, its level dropped to 60–70% and then gradually decreased in the depuration phase. Fluoxetine and paroxetine were not accumulated inside the protozoan cells, and their BCF values during the uptake phase never exceeded 1000 L/kg, while for mianserin and sertraline, the BCF values reached much higher at 4939 and 34,092 L/kg, respectively. The U.S. Environmental Protection Agency has established a BCF ranging from 100 to 1000 L/kg to indicate a medium concern for bioaccumulation [13]; compounds with BCF > 1000 L/kg are considered to be highly bioaccumulating.

The bioaccumulation of SSRIs has been reported in invertebrates and fish by many authors [17,32,33,34], and the results varied depending on the species. The BCF for sertraline calculated by Grabicova et al. [17] for *E. octoculata* and *Hydropsyche* sp. was higher than 2000 L/kg, while Du et al. [32] found that the BCF value for *Planorbid* sp. was only 990 L/kg. These values were an order of magnitude lower than our results obtained for *S. ambiguum*. The largest spread of results was published for fluoxetine. The value close to our value was obtained by Franzellitti et al. [33] in the marine mussel *Mytilus galloprovincialis*; after seven days of treatment at the concentrations of 30 and 300 ng/L, the BCF ranged from 200 to 800 L/kg. A higher value of 3000 L/kg was reported by Du et al. [32] for *Planorbid* sp. In contrast, Meredith-Williams et al. [34] obtained quite different BCF values of 185,900 L/kg and 1387 L/kg in freshwater shrimp (*Gammarus pulex*) and the water boatman (*Notonecta glauca*), respectively. According to these authors, the 2–3 orders of magnitude higher BCF values for fluoxetine in *G. pulex* resulted from the limited depuration in these animals. Our results (Figure 2 and Figure 3) also indicate low depuration of the tested pharmaceuticals from *S. ambiguum*. In the most cases, after transferring the protozoa to a fresh medium, the intracellular concentration decreased only 2–3 times. The differences in the degree of uptake across the different organisms may be due to differences in the mode of respiration, behavior, and pH of the test system. Moreover, the BCF values are reduced as organism size increases and increase with increasing lipid content [34,35]. However, Rubach et al. [36] found no relationship between lipid content and chlorpyrifos uptake in all 15 species of fish they tested. Lipophilicity is the most often used criterion for predicting the bioaccumulation potential. According to European guidelines on environmental risk assessment of medicinal products for human use [37], all drug substances with log P > 4.5 should be considered to be potentially persistent and should be screened for bioaccumulation; however, OECD uses lower criteria of only log P > 3 [38]. Based on the calculated log P values, Howard and Muir [13] classified sertraline, fluoxetine, and paroxetine as potentially bioaccumulative. However, at neutral pH, the log D values are much lower than log P values (Table 1), and this can explain such low BCF values for fluoxetine (log D: 1.23–1.81) and paroxetine (log D: 0.01–0.61). Grabicova et al. [17] showed that the antidepressive drug citalopram tended to accumulate in organisms, and the extent of accumulation was equivalent to the extent of metabolic transformation and removal from the body.

After transferring *S. ambiguum* to a clean solution, very slow elimination was observed, and the drugs were detected inside the cells at concentrations up to 11,000 higher than that in the water phase (Figure 3 and Table A3). This indicates that the protozoa were unable to excrete the accumulated antidepressants. The bioaccumulation of drugs in subcellular organelles may eventually result in phospholipidosis and alkalinization of the lysosomes [39]. Two mechanisms are responsible for the accumulation of the basic amphiphilic compounds in cells: binding to phospholipids and lysosomal trapping [40]. The cell membrane and membranes of cellular organelles are permeable to non-ionized compounds [39]. The most acidic pH of protozoa food vacuoles ranges between 3.5 and 4.0. In these conditions, all the tested antidepressants became protonated and cannot pass through the membrane back to the cytosol, which may result in their accumulation within the lysosomes [39]. This phenomenon is called lysosomotropism and has been found in different mammalian cells [39,40,41]. However, to the best of our knowledge, it has not yet been studied in protozoa. The degree of ion trapping depends on membrane permeability, the pH gradient between the cytosol and lysosome, and physicochemical properties of the compound such as pKa [41]. In our present study, vacuolization of the protozoan cells was observed after six days of incubation with the highest tested concentration of sertraline (100 μg/L) (date not presented). This suggests an effect of the drug on vacuole membrane; however, this hypothesis needs to be confirmed in future research.

### 2.3. Biotransformation

To evaluate biotransformation, the protozoan *S. ambiguum* was incubated with the antidepressant solution (100 μg/L) in darkness for two days. The Orbitrap™ high-resolution UPLC-MS/MS was used to determine the potential metabolites of the antidepressants in both medium and the protozoan cell. The tentative metabolites of the antidepressant were detected by Compound Discoverer Software (Thermo Fisher Scientific).

The tests were performed twice, and the relative area of the chromatogram peaks are presented in Table 2. The chromatograms of the tested samples were compared to that of the control samples. The peaks observed in two replicates of the samples and not visible in two controls were shown. The predicted transformation products and the difference between the measured and theoretical mass are given. As controls, the antidepressant solutions without the protozoa were incubated under the same conditions. No transformation products were observed in the control samples (data not presented), which confirms the previous findings that these compounds are stable in the aquatic environment [42,43]. Derivatives of only two drugs (fluoxetine and paroxetine) were detected in the protozoa homogenates, whereas four to six transformation products were observed in aquatic media for each antidepressant. The very low levels inside the protozoan cells may be caused by the method of sample preparation. Because of their very low volume, the cell homogenates were analyzed without any enrichment techniques, while the medium was concentrated 100-fold by passing it through Hydrophilic-Lipophilic Balance (HLB) cartridges. The lack of metabolites inside the cells could also be caused by their better solubility in water, high elimination rate from the cells, and lower bioconcentration in the cells than those of the parent compounds.

Five mianserin derivatives were observed in the tested samples, and these were N-demethylation and oxidation products (Table 2). The major mianserin metabolites that are formed in the liver in humans are N-desmethylmianserin, 8-hydroxymianserin and mianserin N-oxide (www.drugbank.ca). Similar products, formed probably by oxidation and oxidative desmethylation, were observed for sertraline, but not fluoxetine (Table 2). Because of the low abundance of these compounds, it was not possible to confirm their structure by fragmentation. Three main sertraline metabolites have been reported in humans: desmethylsertraline, sertraline ketone and sertraline N-carbamoyl glucuronide [44]. In humans, fluoxetine and sertraline are mainly metabolized to N-desmethyl products, which retain their pharmacological activity [18]. N-desmethyl metabolites were also found in aquatic organisms. Silva et al. [18] presented several findings on the occurrence of norfluoxetine and norsertraline in many freshwater fish. These metabolites are more stable than their parent compounds and less polar; thus, their levels in many cases were higher than those of their parent compounds, especially in the liver and brain. However, the authors did not provide the source of these metabolites in aquatic organisms. In organisms collected from the environment, the most probable source of these compounds was the accumulation of metabolites of human origin. Only laboratory tests can prove the occurrence of biotransformation processes in aquatic organisms. Rodriguez et al. [45] detected residual norsertraline in crab cultures incubated with sertraline for two days. Chu et al. [46] found increased concentrations of norfluoxetine in fish incubated with fluoxetine. The mussel *M. galloprovincialis* was exposed to a nominal concentration of fluoxetine (75 ng/L) for 15 days [47]. The authors observed that the concentration of fluoxetine and norfluoxetine increased from 2.53 and 3.06 ng/g dry weight after 3 days up to 9.31 and 11.65 ng/g after 15 days, respectively. These results suggest that fluoxetine accumulated in mussel tissues is likely to be metabolized into norfluoxetine with the increase in the time of exposure.

In humans, paroxetine is metabolized to paroxetine catechol, which is methylated and conjugated into second phase metabolites [42,48]. Cleavage of the paroxetine is also possible, which leads to the formation of the metabolite with a molecular mass of 209 Da [48]. The latter compound was also observed in our studies (Table 2).

Two identical derivatives of SSRIs were observed, which resulted from the addition of CO and C15H22O (Table 2). To the best of our knowledge, such transformation products have not been described either for humans or for aquatic organisms. Their structures were not proposed in the current study because of their very low abundance to perform fragmentation studies. However, this will be the subject of future studies.

### 2.4. Ecological Implications

The presence of pharmaceuticals in the environment, with a focus on their presence in water, is a potentially major problem with consequences such as toxicity and/or persistence that have not yet been fully understood. Simultaneously, studies involving topical exposure of protozoa to pharmaceuticals in the aquatic environment are very limited [49]. However, protozoa, next to bacteria, constitute the main group of organisms in activated sludge in WWTP, and they are involved in the removal of pollutants from waste-water [50] and in the freshwater self-purification process. Hence, they could have a significant role in removing drugs from the aqueous phase and in their transfer to higher trophic levels. Considering that neuroactive drugs are one of the most ecotoxic pharmaceuticals and that their removal efficiency depends on the condition of conventional activated sludge in WWTP, it is extremely important to know the mechanisms that enable the functioning of protozoa in such conditions and the potential for recovery after contamination. Acute toxicity results obtained in this study were two orders of magnitude higher than the SSRIs concentrations reported in environmental samples. Thus, it can be concluded that the tested antidepressants are unlikely to be toxic to the aquatic protozoa. On the other hand, according to our research and literature review, the SSRIs have been accumulated in biota, and long-term toxic effects cannot be excluded. Thus, future research should be focused on analyzing the transmission of toxic substances, e.g., pharmaceuticals accumulated in vacuoles, and/or their effects on the next generations of organisms and on the next links in the trophic chain.

## 3. Materials and Methods

### 3.1. Reagents

Standards of fluoxetine (FLU) and mianserin (MNS) as well as internal standards (IS, nortryptyline and doxepin) were obtained from Sigma-Aldrich (Poznań, Poland), while paroxetine (PAR) and sertraline (SER) were gifts from the National Drug Research Institute, Warsaw, Poland. All the drugs were of high purity grade (>90%). The standard stock solutions of all compounds were prepared in methanol at concentrations of 1 mg/mL and stored at −20 °C. Working solutions were prepared ex tempore by dilution of the stock solutions with the culture medium. IS working solution (500 ng/mL) was prepared ex tempore by dilution of the stock solution with acetonitrile. The solvents, namely HPLC gradient grade methanol, MS grade acetonitrile (LiChrosolv) and formic acid 98%, were provided by Merck (Darmstadt, Germany). Ultrapure water was obtained from a Millipore water purification system (Milli-Q water). The pH-dependent octanol-water partition coefficients (log D) were calculated with logD predictor (https://disco.chemaxon.com/apps).

### 3.2. Toxicity Assay

Acute toxicity was determined according to the Spirotox assay procedure [1]. Briefly, the assay was performed in 24-well polystyrene microplates. Five 2-fold dilutions were prepared directly in the multi-well plate. Each well contained 1 mL of the test solution and 10 protozoan cells. The microplates were incubated at 25 °C in darkness. Toxic effects (lethality, sublethal responses such as shortening, bending of the cell, immobilization) were noted after 1, 2, and 7 days of incubation. LC_50_, EC_50_ and EC_20_ values were calculated on the basis of lethal response (L) and all toxic effects (lethal and sublethal) (E), respectively. The toxicity values were expressed in mg/L on the basis of the initial concentrations of the tested compounds. The LC_50_, EC_50_ and EC_20_ values were determined by graphical interpolation of test response versus toxicant concentration (log scale) [3]. As the diluent and control, Tyrod solution [1] buffered with NaH_2_PO_4_ and Na_2_HPO_4_ (50 mM) was used. The toxicity of each compound was tested at pH 6.0; 6.5, and 7.4. All tests were performed in quadruplicate.

### 3.3. Bioaccumulation Test

The experiments were carried out in 250 mL glass beakers filled with 200 mL of sample or control. As a diluent and control, an inorganic medium (Tyrod solution) was used, with pH adjusted to 7.4. The bioaccumulation experiments were performed for the individual drugs: fluoxetine, sertraline, paroxetine, and mianserin (10, 25 and 100 µg/L). The experimental scheme is presented in Table 3. A total of 1000 protozoan cells were added to each beaker, and the beakers were incubated for 6 days (144 h) at 25 °C in darkness. A total of 100 protozoan cells were subsampled from each test beaker after 2 h and after 1, 2, and 6 days of incubation. Simultaneously, 1 mL of water from each sample was taken for chemical analysis. After 6 days, the protozoa (approximately 500) were transferred to a new glass beaker with the fresh Tyrod solution for testing the depuration of the accumulated drugs. Next, 100 protozoan cells and 1 mL of water were subsampled after 1, 2, and 6 days of incubation. The test was performed in duplicate.

Quantitative analyses were performed using Agilent 1260 Infinity (Agilent Technologies, Santa Clara, CA, USA), equipped with a degasser, a thermostated autosampler, a binary pump, and connected in series to a QTRAP^®^4000 (AB SCIEX, Framingham, MA, USA) equipped with a Turbo Ion Spray source operated in the positive mode. The curtain gas, ion source gas 1, ion source gas 2 and collision gas (all high purity nitrogen) were set at 35 psi, 60 psi, 40 psi and ‘medium’ instrument units, respectively, and the ion spray voltage and source temperature were set at 5000 V and 600 °C, respectively. Chromatographic separation was achieved with a Kinetex RP-18 column (100 mm, 4.6 mm, particle size 2.6 µm) supplied by Phenomenex (Torrance, CA, USA). The column was maintained at 40 °C at the flow rate of 0.5 mL/min. The mobile phases consisted of HPLC grade water with 0.2% formic acid as eluent A and acetonitrile with 0.2% formic acid as eluent B. The gradient (%B) was as follows: 0 min, 10%; 1 min, 10%; 8 min, 90%; 9 min, 90%. The volume of injection was 10 µL. The target compounds were analyzed in the multiple reaction monitoring (MRM) mode (Table A4) by monitoring two transitions between the precursor ion and the most abundant fragment ions for each compound.

Preparation of *S. ambiguum* samples for HPLC analysis involved mixing 50 µL of sample (100 protozoan cells + medium) with IS (50 µL) and acetonitrile (100 µL). The samples were vortexed (10 min), placed for 10 min in a freezer (at −20 °C) and then centrifuged (5 min at 10,000× *g*). The supernatant (150 µL) was mixed with 375 µL of water and transferred to the autosampler vial. The concentration of pharmaceuticals in organisms was calculated using the measured concentration of the pharmaceutical in the medium and the volume of *S. ambiguum*. An average volume of 100 cells of *S. ambiguum* was 0.50 µL. The preparation of medium samples for HPLC analysis involved centrifugation (10 min at 10,000× *g*), mixing with the IS (9:1) and transferring to vials. No clean up procedure was used.

The validation was performed according to the European Medicines Agency guideline [37]. For *S. ambiguum* extracts, two linearity ranges were selected: 1–100 µg/L and 50–10,000 µg/L of homogenate. For medium samples, the linearity was selected as 0.2–100 µg/L. The coefficients of determination for curves was above 0.99. All validation experiments (accuracy, precision, variation of the relative matrix effect and stability) met the European Medicines Agency (EMEA) acceptance criteria [51].

The concentration of the tested antidepressants in *S ambiguum* was expressed as µg/g assuming the density of the organism as 1 g/mL. The bioconcentration factor was calculated by dividing the substance concentration in organisms to the concentration in the medium and was expressed as L/kg.

### 3.4. Analysis of Biotransformation of Drugs

The biotransformation of the drugs by the protozoa was analyzed for the four antidepressants: fluoxetine, mianserin, paroxetine, and sertraline. The test beakers were prepared in a manner similar to that for the bioaccumulation experiment. However, only one concentration (100 µg/L) of the drug was tested, and no depuration phase was performed. Concomitant with the tested sample, two control samples were incubated: the abiotic degradation control containing only the same concentration of the tested pharmaceutical (described as “drug control”) and the organism control containing only protozoa. After 2 days of incubation in darkness, 500 protozoan cells in 100 µL of medium were transferred to the Eppendorf tube, and 200 µL of acetonitrile was then added. Samples were vortexed (10 min), placed for 10 min in the freezer (at −20 °C) and centrifuged (5 min at 10,000× *g*). The supernatant (150 µL) was mixed with 375 µL of water and transferred to the autosampler vial. Furthermore, 100 mL of medium was sampled at the end of experiment and poured into the preconditioned Oasis HLB (Waters) *spe* cartridge (30 mg). The analytes were eluted with 2 × 3 mL of methanol. The methanol was evaporated under the stream of nitrogen, and the extract was reconstituted with 1 mL of acetonitrile:water (1:9, *v*/*v*). The analysis of transformation products was performed with Ultra High Performance Liquid Chromatography (UHPLC) Dionex Ultimate 3000 with a Q-Exactive hybrid quadrupole-orbitrap mass spectrometer system. Heat electrospray ionization (HESI) was operated in the positive mode. Full MS scans were acquired over *m*/*z* 75–1100 range with the resolution of 70,000 (*m*/*z* 200). Standard mass spectrometric conditions for all experiments were as follows: spray voltage: 3.5 kV; sheath gas pressure: 60 arb; aux gas pressure: 20 arb; sweep gas pressure: 0 arb; heated capillary temperature: 320 °C; loop count: 3; isolation window: *m*/*z* 3.0; and dynamic exclusion: 6.0 s. Chromatographic separation was achieved using a Kinetex RP-18 column (100 mm × 4.6 mm, 2.6 µm) supplied by Phenomenex and equipped with a security guard. The column was maintained at 40 °C at the flow rate of 0.3 mL/min. The mobile phases consisted of HPLC grade water with 0.1% formic acid as eluent A and acetonitrile with 0.1% formic acid as eluent B. The gradient (%B) was as follows: 0 min–10%; 1.5 min–10%; 7.0 min–90%; 12 min–90%. The volume of injection was 10 µL.

All the chromatograms obtained in the biotransformation experiments were integrated with Compound Discoverer Software. The area of the peaks obtained for the sample (protozoa in the drug solution) was divided by the area of the corresponding peaks of the control (protozoa in the medium). Similarly, the area of the peaks obtained for the drug control was divided by the area of the corresponding peaks of the medium. Thus, three values were obtained: tested medium, extract from the protozoan cells, and control medium.

## 4. Conclusions

We successfully performed a laboratory experiment designed to obtain comprehensive results for acute toxicity, bioconcentration, and biotransformation by determining the biological activity of four antidepressants on the protozoan *S. ambiguum*. The tested compounds were acutely toxic to *S. ambiguum*, and moreover, sublethal effects quickly became lethal ones. Sertraline was the most toxic among the studied antidepressants. However, the toxic effects occur at concentrations at least two orders of magnitude higher than those determined in effluents and freshwaters. Thus, it can be concluded that the tested antidepressants are unlikely to represent a risk to the aquatic protozoa. The results also showed the relationship between pH and toxicity, which has two consequences. First, the pH of the water should be more strictly defined in the aquatic toxicity guidelines to prevent high inter- and intra-laboratory variability of the results. Second, pH of the water and effluent should be considered in the environmental risk assessment, especially for ionizable compounds.

On the basis on the bioconcentration tests, it can be concluded that uptake and elimination kinetics vary greatly between the tested pharmaceuticals. The highest BCF value was obtained for sertraline and mianserin, but different bioaccumulation scenarios can be observed for each pharmaceutical and for each concentration. Our results also indicate that the protozoan cells were unable to excrete the accumulated antidepressants. We suspect that the main reason of the toxic effects and high bioaccumulation ratio were the interactions between the tested drugs and lysosomal membrane phospholipids, which lead to vacuolization. Thus, future research should focus on analyzing the transmission of antidepressants accumulated in vacuoles and/or their effects on the next generations of organisms.

For the first time, the research for the biotransformation products of antidepressants were conducted in the protozoa. However, because of the low abundance of possible biotransformation products, their structure could not be elucidated. This part of the present work revealed a potential for further investigation of pharmaceutical metabolism in protozoa exposed to drugs under natural conditions.

## Figures and Tables

**Table 1:** Physicochemical characteristics of the tested antidepressants.

	Fluoxetine	Paroxetine	Sertraline	Mianserin
	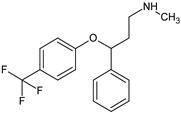	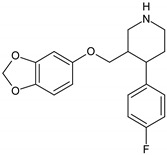	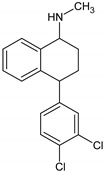	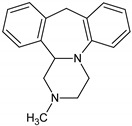
pKa	9.80	9.77	9.85	6.92
Log P	4.09	3.15	5.06	3.83
Log D; pH = 6.0	1.23	0.01	2.17	2.38
Log D; pH = 6.5	1.33	0.11	2.17	2.84
Log D; pH = 7.4	1.81	0.61	2.23	3.50

pKa: acid dissociation constant obtained from www.drugbank.ca; Log P: Octanol-water partition coefficient obtained from www.drugbank.ca; Log D: pH-dependent octanol-water partition coefficient calculated with logD predictor (KLOP algorithm, MarvinSketch 15.2.23. software (https://disco.chemaxon.com/apps).

**Table 2:** Biotransformation of the tested antidepressants by *S. ambiguum*. Relative abundance of the compounds in the protozoan *S. ambiguum* and in the medium after two days incubation of the protozoans with the parent compounds. The test was performed in duplicate.

[M/z]	Molecular Weight	Delta [ppm]	RDB	RT [min]	Formula	Relative to the Parent Compound	Area of the Chromatographic Peak			
							Medium		*S. ambiguum*	
**264.1626**	**264.1621**	**2.0**	**10.0**	**10.13**	**C18H20N2**	**Mianserin**	**25,474**	**31,693**	**32,427**	**28,085**
294.1365	294.1363	0.7	11.0	11.16	C18H20N2O2	+O2	48	59	-	-
280.1579	280.1570	3.1	10.0	10.23	C18H20N2O	+O	478	324	-	-
278.1424	278.1414	3.5	11.0	12.34	C18H18N2O	+O; -H2	180	351	-	-
266.1416	266.1414	0.6	10.0	11.12	C17H18N2O	+O; -C H2	12	10	-	-
250.1474	250.1465	3.7	10.0	12.34	C17H18N2	-C H2	25	57	-	-
**309.1342**	**309.1335**	**2.3**	**8.0**	**10.62**	**C17H18F3NO**	**Fluoxetine**	**20,004**	**33,119**	**9485**	**8925**
527.3015	527.3006	1.7	12.0	11.99	C32H40F3NO2	+C15 H22 O	314	131	-	-
372.1786	372.1781	1.4	6.5	10.56	C19H25F3NO3	+C2 H7 O2	21	11	4	-
365.1603	365.1597	1.7	9.0	13.76	C20H22F3NO2	+C3 H4 O	12	15	-	-
351.1445	351.1441	1.1	9.0	13.26	C19H20F3NO2	+C2 H2 O	74	103	-	-
337.1290	337.1284	1.8	9.0	13.18	C18H18F3NO2	+C O	200	189	-	-
147.1048	147.1043	3.5	5.0	10.62	C10H13N	-C7 H5 F3 O	2360	1179	338	324
**329.1423**	**329.1422**	**0.3**	**10.0**	**10.33**	**C19H20FNO3**	**Paroxetine**	**12,009**	**405**	**1965**	**2588**
547.3101	547.3092	1.6	14.0	11.77	C34H42FNO4	+C15 H22 O	140	12	-	-
371.1537	371.1527	2.6	11.0	12.83	C21H22FNO4	+C2 H2 O	157	95	-	-
357.1373	357.1371	0.6	11.0	12.72	C20H20FNO4	+CO	201	125	-	-
343.1578	343.1578	0.0	10.0	10.39	C20H22FNO3	+CH2	102	50	52	44
209.1216	209.1210	3.0	5.0	3.51	C12H16FNO	-C7 H4 O2	38	24	-	-
**305.0733**	**305.0733**	**0.0**	**9.0**	**10.66**	**C17H17Cl2N**	**Sertraline**	**45,092**	**14,502**	**46,409**	**37,880**
523.2410	523.2403	1.3	13.0	12.45	C32H39Cl2NO	+C15 H22 O	134	34	-	-
333.0687	333.0682	1.4	10.0	14.11	C18H17Cl2NO	+CO	85	186	-	-
321.0688	321.0682	2.0	9.0	10.05	C17H17Cl2NO	+O	36	95	-	-
305.0374	305.0369	1.7	10.0	14.29	C16H13Cl2NO	+O; -C H3	46	15	-	-

**Table 3:** Bioaccumulation experiment with protozoan *S. ambiguum*. During the sampling 1 mL of medium and 100 protozoans were collected for chemical analyses.

Preparation:		Accumulation Phase				Depuration Phase		
	0 h	2 h	1 d	2 d	6 d	1 d	2 d	6 d
Medium	+200 mL	−1 mL	−1 mL	−1 mL	−1 mL	−1 mL	−1 mL	−1 mL
*S. ambiguum*	+1000	−100	−100	−100	−100	−100	−100	−100

## Appendix A

**Table A1:** Toxicity of the selected antidepressants in the Spirotox assay [mg/L].

Fluoxetine		pH = 6.0	pH = 6.5	pH = 7.4
1 d	LC_50_	5.13 ± 0.41	2.78 ± 0.07	1.73 ± 0.17
	EC_50_	4.75 ± 0.23	2.32 ± 0.24	1.68 ± 0.19
	EC_20_	2.03 ± 0.82	1.16 ± 0.14	1.21 ± 0.04
2 d	LC_50_	4.06 ± 0.10	1.78 ± 0.46	1.52 ± 0.16
	EC_50_	2.88 ± 0.38	1.49 ± 0.14	1.39 ± 0.13
	EC_20_	1.72 ± 0.51	0.98 ± 0.16	1.06 ± 0.11
7 d	LC_50_	2.03 ± 0.55	0.99 ± 0.16	1.35 ± 0.07
	EC_50_	1.94 ± 0.53	0.96 ± 0.16	1.05 ± 0.22
	EC_20_	0.86 ± 0.15	0.58 ± 0.07	0.70 ± 0.13
**Mianserin**		**pH = 6.0**	**pH = 6.5**	**pH = 7.4**
1 d	LC_50_	6.43 ± 0.16	3.95 ± 1.02	2.64 ± 0.50
	EC_50_	6.27 ± 0.09	3.88 ± 1.11	2.35 ± 0.33
	EC_20_	3.34 ± 0.76	2.68 ± 0.67	1.58 ± 0.40
2 d	LC_50_	5.47 ± 0.24	3.51 ± 1.28	2.20 ± 0.44
	EC_50_	4.80 ± 0.41	3.34 ± 1.23	1.55 ± 0.18
	EC_20_	2.90 ± 0.25	2.70 ± 0.59	1.16 ± 0.08
7 d	LC_50_	3.56 ± 0.76	1.34 ± 0.04	1.42 ± 0.07
	EC_50_	2.75 ± 0.57	1.20 ± 0.18	1.32 ± 0.12
	EC_20_	2.05 ± 0.23	0.66 ± 0.04	1.01 ± 0.18
**Paroxetine**		**pH = 6.0**	**pH = 6.5**	**pH = 7.4**
1 d	LC_50_	4.53 ± 0.55	1.40 ± 0.06	1.17 ± 0.29
	EC_50_	3.15 ± 1.68	1.37 ± 0.03	1.15 ± 0.29
	EC_20_	2.46 ± 0.46	1.05 ± 0.05	0.85 ± 0.24
2 d	LC_50_	2.55 ± 0.75	1.27 ± 0.16	1.15 ± 0.30
	EC_50_	2.07 ± 0.73	1.24 ± 0.17	1.10 ± 0.29
	EC_20_	1.71 ± 0.30	0.77 ± 0.45	0.85 ± 0.24
7 d	LC_50_	1.44 ± 0.21	0.81 ± 0.31	0.77 ± 0.10
	EC_50_	1.32 ± 0.13	0.76 ± 0.26	0.74 ± 0.06
	EC_20_	1.12 ± 0.02	0.44 ± 0.16	0.58 ± 0.02
**Sertraline**		**pH = 6.0**	**pH = 6.5**	**pH = 7.4**
1 d	LC_50_	0.85 ± 0.09	0.47 ± 0.11	0.41 ± 0.05
	EC_50_	0.73 ± 0.14	0.42 ± 0.08	0.36 ± 0.01
	EC_20_	0.46 ± 0.18	0.28 ± 0.04	0.29 ± 0.01
2 d	LC_50_	0.49 ± 0.10	0.33 ± 0.06	0.35 ± 0.01
	EC_50_	0.39 ± 0.12	0.31 ± 0.06	0.35 ± 0.02
	EC_20_	0.19 ± 0.03	0.22 ± 0.08	0.29 ± 0.01
7 d	LC_50_	0.33 ± 0.08	0.18 ± 0.04	0.27 ± 0.03
	EC_50_	0.28 ± 0.05	0.17 ± 0.03	0.23 ± 0.04
	EC_20_	0.20 ± 0.05	0.13 ± 0.03	0.15 ± 0.01

**Table A2:** Concentration of the tested antidepressants in *S. ambiguum* in µg/g.

Fluoxetine	10 µg/L		25 µg/L		100 µg/L	
Sampling:	1	2	1	2	1	2
0.083 d	1.02	1.80	1.20	4.70	2.95	14.66
1 d	1.27	3.36	2.94	11.04	10.02	29.54
2 d	2.17	2.71	3.57	6.65	15.45	30.51
6 d	3.66	6.65	4.65	10.91	12.82	28.65
7 d	1.32	2.27	1.52	3.37	5.75	15.46
8 d	1.09	2.30	1.73	3.13	5.08	11.77
12 d	1.61	2.05	1.89	2.88	3.15	9.07
**Mianserin**	**10 µg/L**		**25 µg/L**		**100 µg/L**	
**Sampling:**	**1**	**2**	**1**	**2**	**1**	**2**
0.083 d	19.88	18.37	81.76	76.13	110.04	272.27
1 d	48.43	40.66	148.83	107.07	229.05	275.21
2 d	54.28	60.35	66.51	132.7	256.15	259.24
6 d	30.32	42.43	72.79	72.59	146.31	239.26
7 d	16.39	13.88	24.21	24.71	51.36	52.78
8 d	17.68	12.49	21.55	24.79	56.04	63.88
12 d	11.29	8.23	21.29	15.04	46.07	42.08
**Paroxetine**	**10 µg/L**		**25 µg/L**		**100 µg/L**	
**Sampling:**	**1**	**2**	**1**	**2**	**1**	**2**
0.083 d	0.72	3.01	0.61	6.75	1.27	22.33
1 d	1.89	3.36	2.36	7.33	32.43	31.86
2 d	0.84	3.85	3.03	9.46	17.45	33.26
6 d	2.49	4.34	4.50	7.78	13.46	30.14
7 d	0.45	<LOD	1.19	<LOD	7.44	8.12
8 d	0.40	<LOD	1.40	<LOD	6.90	6.38
12 d	1.82	<LOD	1.85	<LOD	4.11	4.42
**Sertraline**	**10 µg/L**		**25 µg/L**		**100 µg/L**	
**Sampling:**	**1**	**2**	**1**	**2**	**1**	**2**
0.083 d	12.01	32.52	54.34	97.41	194.84	409.65
1 d	50.14	112.44	179.26	377.38	900.06	1058.73
2 d	58.24	135.65	161.30	290.20	890.58	935.23
6 d	75.49	159.08	212.90	371.61	673.24	832.82
7 d	22.06	65.37	94.85	255.51	574.47	645.00
8 d	28.55	66.59	116.04	227.17	641.16	619.90
12 d	27.22	68.21	120.04	286.69	525.78	664.49

**Table A3:** Bioconcentration factor of the tested antidepressants in *S. ambiguum* in L/kg. The test was performed in duplicate.

Fluoxetine	10 µg/L		25 µg/L		100 µg/L	
Sampling:	1	2	1	2	1	2
0.083 d	123	202	81	248	39	359
1 d	157	359	217	585	129	696
2 d	306	283	238	392	191	786
6 d	387	565	328	473	255	600
7 d	4079	11,267	4358	4859	11,122	5738
8 d	3439	9045	5259	4108	10,776	4228
12 d	1000	1065	3500	1779	4256	2257
**Mianserin**	**10 µg/L**		**25 µg/L**		**100 µg/L**	
**Sampling:**	**1**	**2**	**1**	**2**	**1**	**2**
0.083 d	2320	1445	3666	2450	1395	2357
1 d	6092	3366	7190	4033	3138	2621
2 d	7497	5721	3276	4666	3318	2137
6 d	4381	4939	3772	3430	1885	2973
7 d	32,774	27,757	16,138	6648	10,271	4008
8 d	35,358	24,976	14,364	7201	11,208	4854
12 d	22,287	16,464	14,191	5169	9214	4004
**Paroxetine**	**10 µg/L**		**25 µg/L**		**100 µg/L**	
**Sampling:**	**1**	**2**	**1**	**2**	**1**	**2**
0.083 d	130	267	54	221	18	171
1 d	389	280	214	247	523	252
2 d	189	290	281	324	349	277
6 d	524	357	482	281	293	286
7 d	905		794		1487	6770
8 d	806		934		1379	5148
12 d	3649		1234		823	3179
**Sertraline**	**10 µg/L**		**25 µg/L**		**100 µg/L**	
**Sampling:**	**1**	**2**	**1**	**2**	**1**	**2**
0.083 d	1092	2374	2394	3516	1203	5383
1 d	6803	14,603	17,574	30,933	18,037	26,468
2 d	7745	22,238	18,351	20,582	13,352	21,649
6 d	10,573	30,015	18,643	34,092	12,033	16,362
7 d	8261	19,810	18,781	35,987	19,674	26,653
8 d	10,573	21,482	22,532	32,453	21,301	25,616
12 d	10,800	20,669	20,140	34,962	18,358	25,856

**Table A4:** MS/MS optimized conditions for the analyzed compounds and internal standards.

*[M + H]^+^ Ion*	*Quantitative Product Ion*	Compound	*DP*	*CE*	*CXP*	*Qualitative Product Ion*	*CE*	*CXP*
			[V]	[V]	[V]		[V]	[V]
280.1	107.0	Doxepin	71	33	6	235.1	25	14
310.3	44.1	Fluoxetine	56	37	6	148.1	13	12
265.1	208.0	Mianserin	96	31	12	118.0	43	8
264.1	233.1	Nortriptyline	71	23	14	91.1	35	6
330.2	192.1	Paroxetine	51	31	14	70.1	49	4
306.0	158.9	Sertraline	51	35	12	275.0	19	16

*CE*-collision energy; *DP*-declustering potential; *CXP*-collision cell exit potential.
